# Release of DNA from *Dermanyssus gallinae* during the Biting Process

**DOI:** 10.3390/ani12091084

**Published:** 2022-04-22

**Authors:** Nicola Pugliese, Donato Antonio Raele, Antonella Schiavone, Maria Assunta Cafiero, Lucia Potenza, Rossella Samarelli, Elena Circella, Ilaria Vasco, Germana Pennuzzi, Antonio Camarda

**Affiliations:** 1 Dipartimento di Medicina Veterinaria, Università degli Studi di Bari, S.P. per Casamassima, km 3, 70010 Valenzano, Italy; nicola.pugliese@uniba.it (N.P.); antonella.schiavone@uniba.it (A.S.); rossella.samarelli@uniba.it (R.S.); elena.circella@uniba.it (E.C.); 2 Istituto Zooprofilattico Sperimentale della Puglia e della Basilicata, Via Manfredonia, 20, 71121 Foggia, Italy; donatoantonio.raele@izspb.it (D.A.R.); mariaassunta.cafiero@izspb.it (M.A.C.); ilaria.vasco@izspb.it (I.V.); germana.pennuzzi@izspb.it (G.P.); 3 Dipartimento di Scienze Biomolecolari, Università degli Studi di Urbino Carlo Bo, Via A. Saffi, 2, 61029 Urbino, Italy; lucia.potenza@uniurb.it

**Keywords:** *Dermanyssus gallinae*, laying hens, ITS, COI, nuclear DNA, mitochondrial DNA, seminested PCR, real-time PCR, fluorescent in situ hybridization

## Abstract

**Simple Summary:**

Like many hematophagous parasites, the poultry red mite *Dermanyssus gallinae* may release some material during the biting process. This investigation evidenced that small amounts of mite DNA may be found in chicken skin after *D. gallinae* infestation. Since the retrieved DNA is both of nuclear and mitochondrial origin, it is possible to hypothesize that, while biting, the mite releases cellular material.

**Abstract:**

*Dermanyssus gallinae* is a hematophagous ectoparasitic mite that usually infests poultry, but is also known for occasionally attacking other animals and humans. It represents a major problem for poultry systems all over the world, with detrimental effects for both production and animal welfare. Despite the significance of *D. gallinae*, very little is known about the biting process to date. Therefore, this study has aimed to verify if mite DNA is injected into the host skin during the blood meal. Mite DNA has been detected by seminested PCR from infested chicken skin and quantified by real-time PCR. Furthermore, its localization within the host tissue has been checked by fluorescent in situ hybridization. Results showed that a very little amount of *D. gallinae* DNA can be released by mites, suggesting that the latter do not introduce whole or partially destroyed cells into the host, but rather it injects traces of nucleic acids, possibly together with merocrine secretions.

## 1. Introduction

The poultry red mite (PRM) *Dermanyssus gallinae* (De Geer 1778) is a hematophagous ectoparasite that usually infests avian hosts, mainly poultry [1]. A blood meal is necessary for protonymphs and deutonymphs to proceed to the next developmental stages, and for adult females to lay eggs. Larvae do not feed on blood [2].

During the blood meal, protonymphs, deutonymphs, and adults may ingest up to 0.02, 0.05, and 0.2 mg of blood, respectively [3]. Considering that dozens to hundreds of thousands of mites may attack a single host each night in the most severe infestations, their effects on poultry are detrimental in terms of welfare and productivity [4,5,6]. Additionally, *D. gallinae* may bite humans, and people working or living in poultry farms are the most exposed [7]. Infestations have been recorded more frequently in Europe among urban dwellers who did not come into contact with poultry [8]. Attacked humans usually report non-specific dermatitis characterized by small erythematous papules [8,9], with intense pruritus and consequent heavy itching, especially in children [10]. A further risk factor is represented by the association between *D. gallinae* and several pathogens of medical and veterinary interest [11,12,13,14].

Despite the relevance of *D. gallinae* infestation, very little is still known about the biting process and its consequences for hosts. Histological analysis performed on the skin of infested hens revealed inflammatory-like reactions characterized by hyperkeratosis, acanthosis, and small but numerous focal lymphocytic infiltrations under the epidermis, in the connective tissue, and around congested blood vessels in derma [15]. In humans, histological examinations evidenced small epidermal defects with fibrinous exudate and perivascular inflammatory reactions in cutis [16].

The inflammatory response in hens after *D. gallinae* infestation was confirmed by Harrington and colleagues [17], who related continuous infestations to a delayed increase in IgM levels, and a more rapid increase in IgY levels. The same authors also reported that IgY recognized a 60 kDa protein from the mite, and further studies associated the IgY increase with exposure to recombinant proteins derived by CatD-1, CatL-1, and Lgm [18]. On the other hand, *D. gallinae* infestation seems to have minimal or no influence on the levels of cytokine expression [17,19]. The identification of proteins responsible for the inflammatory response is a crucial step in potential vaccine development [20]. In vitro investigations found that some proteins (namely, Dg-CatL-1, Deg-SRP-1, Deg-VIT-1, Deg-HGP-1, and Deg-PUF-1) evoked a significant immune response in chickens [21,22]. More recently, studies have also been undertaken to ascertain the role of another protein, the cysteine protease Deg-CPR-2 [23]. Some authors speculated that immunoglobulin of immunized hosts might block some vital protein of the mite [22], but the actual role of those potential targets needs further investigation, as well as their possible injection route, if any. In particular, it is not clear if mites inoculate saliva, cellular material, or even both during their blood meal.

In light of this, the present study aimed to verify if cellular material from *D. gallinae* is inoculated into the host skin during the bite, by detecting the nuclear and mitochondrial DNA of the mite through a sensitive seminested PCR (snPCR) and fluorescent in situ hybridization (FISH) from the skin of heavily infested hens.

## 2. Materials and Methods

### 2.1. Biological Samples

The samples for the investigation were collected from an industrial laying hen farm that was experiencing a heavy infestation of *D. gallinae*, corresponding to level IV of the Cox’s scale (included in the study by Mul and colleagues [24]) with clusters of mites larger than 1 cm^2^ visible in unprotected sites. Mites were collected from cages and walls, and identified as *D. gallinae* according to the morphological keys of Varma and Baker [25,26]. Prior to further analyses, mites were starved for 7 days to digest the blood they ingested before collection.

At the same time, laying hens found dead were also collected and necropsied.

Similarly, spontaneously dead chickens were collected from another farm, not infested by *D. gallinae*.

After feathers were carefully plucked, two rectangular blocks of skin, each measuring about 4–5 cm^3^ (about 3.5 cm wide, 3.5 cm long, 0.3 cm deep), were collected from the front neck area of three animals from the infested farm (termed A, B, and C) and from the non-infested farm (D). The biological samples included epidermis and derma. The surface of each tissue piece was carefully inspected by the mean of a stereomicroscope to ensure that no mites were still present. After inspection, one out of the two samples from each animal was washed with 4% *w/v* paraformaldehyde and rinsed with sterile distilled water. Total genomic DNA extraction was carried out from 30 mg of tissue from the paraformaldehyde-washed samples by the mean of the GenElute Mammalian Genomic DNA Miniprep kit (Sigma-Aldrich, Milan, Italy) according to the manufacturer’s instructions. Similarly, the same kit was used to purify the total DNA from three aliquots of 100 mites, which were previously ground with sterile pestle and mortar.

The other tissue samples were paraffin-fixed and formalin-embedded.

### 2.2. Seminested PCR

In order to improve sensitivity, a seminested PCR (snPCR) protocol was designed to detect DNA from *D. gallinae*. Briefly, available sequences of the ribosomal internal transcribed spacer (ITS) and the cytochrome oxidase subunit I (COI) of *D. gallinae* were downloaded from GenBank and aligned by the Clustal W algorithm implemented in MEGA X [27]. Primers were manually designed on the basis of the most conserved regions. The reverse primer (ITS2-R) of the first step of the ITS-based snPCR was from Potenza and colleagues [28]. The complete set of primers is listed in Table 1.

A total of 4 μL of DNA solution was used in each first-step PCR reaction. The reaction mixture contained 1 U of Platinum Taq Polymerase (Thermo Scientific, Milan, Italy), 20 mM Tris-HCl, 50 mM Kcl, 1.5 mM MgCl_2_ 0.2 mM of each dNTP, and 25 pmol of each outer primer (ITSDGF/ITS2-R or FCOIDG/RCOIDG) in a final volume of 50 μL. The reactions were performed in a Mastercycler (Eppendorf AG, Hamburg, Germany) using the following thermal cycle: initial denaturation and Taq polymerase activation at 94 °C for 5 min followed by 35 cycles of 94 °C for 30 s, 56.6 °C for 30 s and 72 °C for 1 min, and final extension at 72 °C for 10 min.

The second-step PCR was carried out in a volume of 25 μL, containing 1 U of Platinum Taq polymerase, 20 mM Tris-HCl, 50 mM KCl, 1.5 mM MgCl_2_, 0.2 mM of each dNTP, and 12.5 pmol of each primer (specifically, the pairs were ITSDGF/ITSDGR and FCOIDG/COIDGSN for ITS and COI, respectively). A total of 1 μL of the first-step reaction was used as a template. The thermal profile was as follows: initial denaturation and Taq polymerase activation at 94 °C for 5 min followed by 35 cycles of 94 °C for 30 s, 55.6 °C for 30 s and 72 °C for 40 s, with a final extension at 72 °C for 10 min.

Each assay was carried out in duplicate and a negative control without DNA was added. The amplification results were visualized following agarose gel electrophoresis at 7.5 V/cm and staining with ethidium bromide 0.5 μg/mL. The images were digitalized by the mean of a GelDoc-It Imaging System (UVP, Upland, US).

### 2.3. Cloning and Nucleotide Sequence Determination

The gathered amplicons from the first-step PCR were purified by using the GenElute PCR Clean-Up kit (Sigma-Aldrich) according to the manufacturer’s instructions, and they were directly sequenced by the Big Dye Terminator and sequence determination was performed through an Applied Biosystem ABI 3100 at the facilities of Bmr Genomics (Padova, Italy). The amplicons from the second-step PCR were gel-eluted by using the GenElute Gel Extraction kit (Sigma-Aldrich) according to the manufacturer’s instructions. Following this, they were cloned in pGEM-T Easy cloning vector (Promega, Milan, Italy) according to the manufacturer’s instructions. Plasmids were purified by using the PureLink Quick Plasmid Miniprep kit (Thermo Scientific), and the nucleotide sequence of the cloned fragments was determined as described above.

The identification of the PCR products was performed through comparison with the sequences in GenBank by BLAST. The nucleotide sequences of the amplicons from the first reaction of the snPCR targeting ITS and COI of isolated *D. gallinae* were submitted in GenBank under the accession numbers KY025551 and KY025552, respectively, while the nucleotide sequences from the first reaction of the ITS-targeting snPCR and the second reaction of the COI-targeting snPCR from chicken skin were submitted under accession numbers KY025553 and KY025554, respectively.

### 2.4. qPCR

In order to quantify the *D. gallinae* DNA in the hens’ tissues, qPCRs were performed, separately targeting ITS and COI. The reactions were performed by using the Power SYBR Green PCR Mastermix (Thermo Scientific) and the primer pairs used in the second step of the snPCRs, namely ITSDFG/ITSDGR and DGCOIF/COIDGSN, according to the selected target. The final concentration of each primer in the reaction mixture was 0.4 μM, and 4 μL of the extracted DNA (about 25 ng of DNA) was used as a template. The reactions were carried out in a StepOne Real-Time PCR System. Tenfold serial dilutions, from 10^−1^ to 10^−7^, of the recombinant plasmids containing the snPCR products from ITS or COI were used as standards. The concentration of the standards was determined by measuring the 260 nm absorbance of the plasmid solution by the NanoDrop 1000 Spectrophotometer (Thermo Scientific). Negative controls were prepared by adding sterile water instead of DNA. All standards, samples, and negative controls were used in triplicate in the same experiment, and the melting curve of the amplification product was also obtained. Thresholds and baselines were automatically assigned by the StepOne software v2.2.2 (Thermo Scientific).

### 2.5. Fluorescent In Situ Hybridization

Longitudinal sections of 5 μm were prepared from formalin-fixed, paraffin-embedded tissues and placed on a microscopy slide. Three sections per block underwent FISH experiments. Sections were deparaffinized by xylene treatment, rehydrated, fixed with 3:1 *v/v* methanol-acetic acid, aged at 65 °C for 30 min, and dehydrated.

A total of 1 μg of the recombinant plasmids containing the first-step PCR products from the ITS and COI regions, respectively, was directly labeled with Cy3-dUTP and FluorX-dUTP, respectively. Labeling was performed by nick translation as previously described [29,30]. The labeled probes were included in the hybridization mixture, which also contained 2X saline sodium citrate solution (SSC), 50% *v/v* formamide, 10% *w/v* dextran sulfate, and 3 mg of DNA from sonicated salmon sperm. Hybridization was carried out with 10 μL of hybridization mixture in a Vysis HyBrite (Abbot Molecular, Rome, Italy) at 65 °C for 5 min and 37 °C for 16 h. Slides were washed three times in high stringency conditions, by incubating them in 2X SSC at 65 °C, and air-dried. Double-stranded DNA was counterstained by 4′,6-diamino-2-phenylindole (DAPI). Digital images were acquired by using a Leica DMRXA2 (Leica, Wetzlar, Germany) epifluorescence microscope equipped with a cooled CCD camera (Princeton Instruments, Planegg, Germany). Cy3, FluorX, and DAPI fluorescence signals were detected with specific filters and recorded separately as grayscale images. Pseudocoloring and merging of images were performed using Adobe Photoshop software (Adobe Systems, San Jose, CA, US). Cy3 (red), FluorX (green), and DAPI (blue) fluorescence signals were converted in red, green, and blue, respectively.

## 3. Results

### 3.1. PCR Detection and Quantification of Mite DNA in Chicken Skin

**Figure 1:**
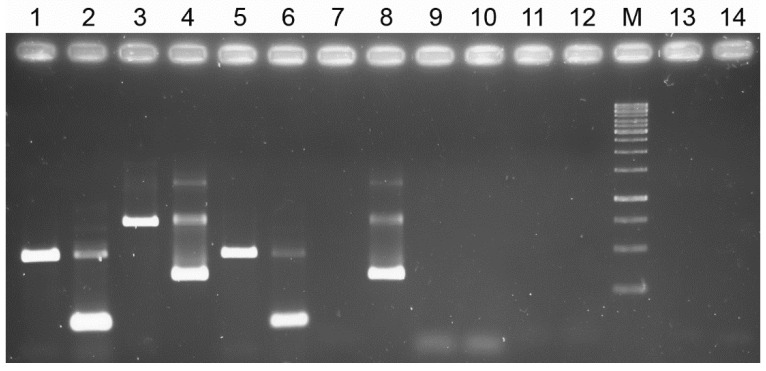
Seminested PCR results from *Dermanyssus gallinae* and chickens’ skin. 1: DNA from *D. gallinae*, primers ITSDGF/ITS2-R; 2: DNA from *D. gallinae*, primers ITSDGF/ITSDGR; 3: DNA from *D. gallinae*, primers FCOIDG/RCOIDG; 4: DNA from *D. gallinae*, primers FCOIDG/COIDGSN; 5: DNA from infested laying hen skin, primers ITSDGF/ITS2-R; 6: DNA from infested laying hen skin, primers ITSDGF/ITSDGR; 7: DNA from infested laying hen skin, primers FCOIDG/RCOIDG; 8: DNA from infested laying hen skin, primers FCOIDG/COIDGSN; 9: DNA from non-infested laying hen, primers ITSDGF/ITS2-R; 10: DNA from non-infested laying hen, primers ITSDGF/ITSDGR; 11: DNA from non-infested laying hen, primers FCOIDG/RCOIDG; 12: DNA from non-infested laying hen, primers FCOIDG/COIDGSN; M: O’GeneRuler 1 kb DNA ladder (Fermentas, Milan, Italy). Band size, from the bottom: 250 bp, 500 bp, 750 bp, 1000 bp, 1500 bp, 2000 bp, 2500 bp; 13: negative control, primers ITSDGF/ITSDGR; 14: negative control, primers FCOIDG/COIDGSN.

The snPCR resulted positive from samples of chicken skin. Specifically, the ITS-targeting snPCR returned an amplicon from both first and second reactions of the snPCR, while for COI, only the second reaction returned an amplicon (Figure 1). The nucleotide sequences of amplicons from chicken skin samples were 100% identical to those from the mite ones, deriving from the same farm the hens were reared.

The nucleotide sequences of ITS and COI were, in turn, 98–99% and 96–100% identical, respectively, to the corresponding sequences from several *D. gallinae* isolates present in GenBank, thus confirming both the identification of mites and the specificity of the snPCR procedure.

**Figure 2:**
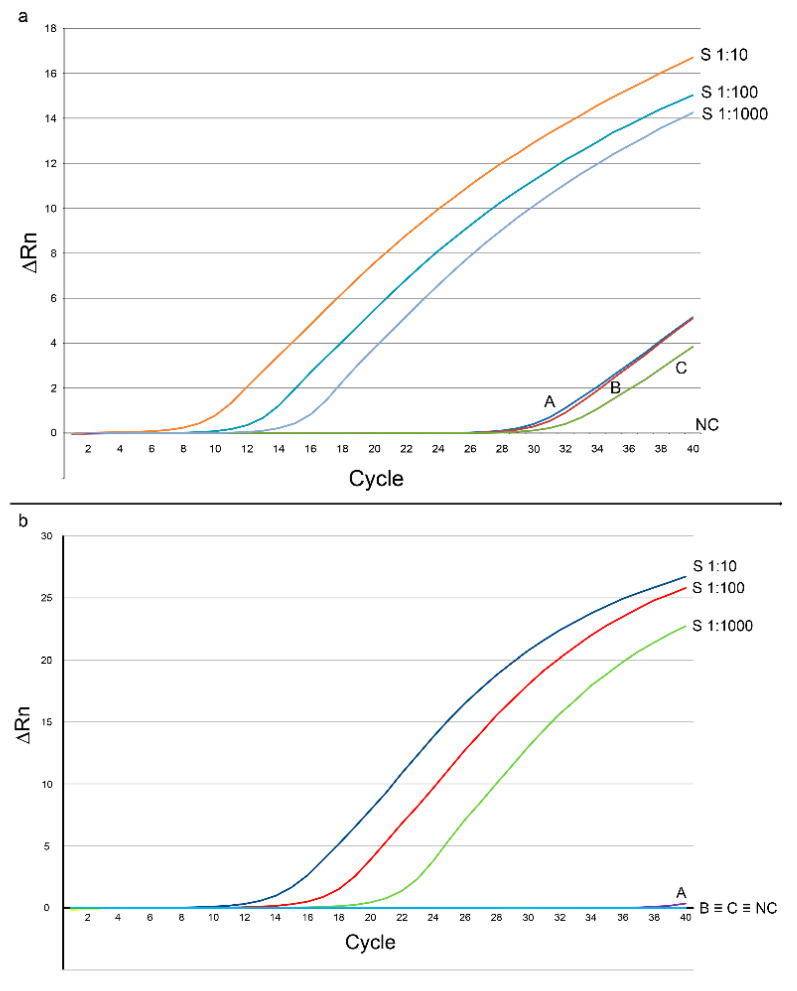
Results of the real-time PCR experiments for the quantification of *Dermanyssus gallinae* ITS (**a**) and COI (**b**) in the laying hen skin. In order to simplify the reading, only one curve out of the three repetitions per sample, and only three standard dilutions have been included. S1:10: standard dilution 1:10; S1:100: standard dilution 1:100; S1:1000: standard dilution 1:1000; A; B; C: DNA from chicken tissue; NC: negative control. ΔRn: reporter signal normalized to the fluorescence of ROX, minus the baseline. The curves from the lower dilutions of the standards have not been reported to avoid readability problems in the figures.

On the other hand, the qPCR only showed an amplification curve when ITS was targeted (Figure 2a), while no amplification signal was evidenced with COI-targeting primers (Figure 2b). A weak amplification curve was observed from sample A, but the melting temperature was 65.18 ± 3.27 °C, significantly different from the melting curve of the standard (75.34 ± 0.10 °C), thus suggesting an aspecific amplification.

From a quantitative point of view, the ITS-targeting qPCR revealed that 76.54 ± 25.65, 27.06 ± 6.91, and 10.00 ± 0.98 copy-numbers of the target were present in 25 ng of DNA from the skin samples A, B, and C, respectively.

### 3.2. Fluorescent in Situ Hybridization

**Figure 3:**
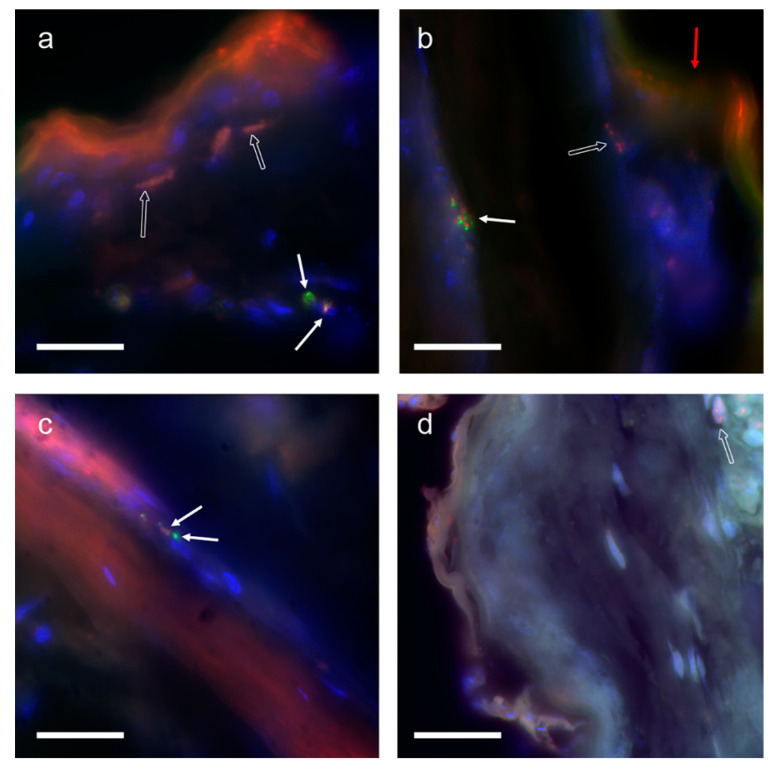
Fluorescent in situ hybridization experiments on skin sections of infested (**a**–**c**) and not-infested chickens (**d**). Figures are in false colors. Red: fluorescent signal from Cy3, labeling the ITS-based probe; green: fluorescent signal from FluorX, labeling the COI-based probe; blue: fluorescent signal from DAPI. White arrows: putative positive signal; empty arrows: putative non-specific signals; red arrow: self-fluorescence of the sample. Magnification bar: 20 μm.

The FISH experiments evidenced only a few clear and well-defined fluorescent signals, unequivocally referable to the specific hybridization of probes to their proper targets. In other cases, the interpretation of signals was uncertain, since they were probably due to aspecific precipitation of fluorescent material (Figure 3).

In one case, the possible presence of an intact mite cell was evident (Figure 3a), as a signal from Cy3 (labeling the ITS probe) was clearly overlapped to a nucleus, close to a signal from FluorX (labeling the COI probe), which might correspond to the cytoplasmic distribution of mitochondria. A similar, but not well defined, signal combination was detected in a slide from sample B (Figure 3c). In this case, the FluorX signal was concentrated in two spots in close proximity to a Cy3 signal.

## 4. Discussion

The gathered results suggest that *D. gallinae* releases a small amount of DNA during the blood meal. It may be noteworthy that the results suggest that the mitochondrial DNA (represented by COI) may be lower in amount than nuclear DNA, whose ITS is a marker. In fact, only the second step of the COI-targeting snPCR returned the expected amplicon, which was yet observed in the first step of the ITS-targeting snPCR. Such a finding was confirmed by qPCR, which only detected and quantified ITS. The low quantity of the target, along with the high sensitivity of snPCR, capable of revealing the presence of very few target copies [31], might account for the apparent discrepancy between snPCR and qPCR, as the former failed in detecting COI.

From a biological point of view, it could be speculated that, for the apparent discordance between *D. gallinae* ITS and COI amount in chickens’ skin, nuclear material may be more stable than mitochondrial. This phenomenon has been observed and described, despite the causes being not completely elucidated. It is possible that specific nucleases might be present in the cell, thus degrading the mitochondrial DNA faster, or that the tight association with the proteinaceous backbone of the nuclear chromosomes is more complex than the mitochondrial one [32]. The FISH experiments seem to confirm such a hypothesis, as the fluorescent signal of the COI-based probe was mainly found in the intact cells, while scattered signals from the ITS-based probe were not always associated with whole cells.

On aggregate, the results infer that, during its blood meal, *D. gallinae* introduces small amounts of its DNA within the host tissue. Considering the low quantity of mite DNA, it appears unlikely that it could come from apocrine or holocrine secretion mechanisms, which imply whole or partial cell disruption, respectively, as seldom described for other arthropods [33,34]. Therefore, it is probable that the mite proteins that elicit the inflammatory reactions in the host [21,22,23,35] are produced by not yet characterized salivary glands, which probably secrete their products in a merocrine fashion.

Finally, it is not negligible that the slight but constant detection of mite DNA from hen skin may be useful for diagnostic purposes, both in animals and humans.

## 5. Conclusions

Considering all of the above, it is possible to affirm that *D. gallinae* releases a detectable amount of DNA while biting the host, and to hypothesize that the DNA retrieved from the infested chicken skin may come from some cellular residual of the mite buccal apparatus that contaminated tissue during the blood meal. The mite DNA might also represent a potential target for future diagnostic applications aimed to confirm suspected dermanyssosis in humans.

## Figures and Tables

**Table 1:** List of primers used in the seminested and real-time PCR.

Primer Name	Nucleotide Sequence	Target	Reference
ITSDGF	ATCCWTTCACTCACKCAGAG	Internal transcribed spacer 1	This study
ITS2-R	GGGGTCGTCACACTTGATTT	Internal transcribed spacer 2	[28]
ITSDGR	GTGAGTACGCGATACRAATYTAG	Internal transcribed spacer 1	This study
FCOIDG	CATTAATATTAACTGCACCTGACA	*Cytochrome oxidase subunit I*	This study
RCOIDG	CCCGTGGAGTGTTGAAATTCA	*Cytochrome oxidase subunit I*	This study
COIDGSN	AAATTGYRGTAATTAAAATAGAYCATG	*Cytochrome oxidase subunit I*	This study

## Data Availability

The nucleotide sequences of the amplicons from the first reaction of the snPCR targeting ITS and COI of isolated D. gallinae were submitted in GenBank under the accession numbers KY025551 and KY025552, respectively, while the nucleotide sequences from the first reaction of the ITS-targeting snPCR and the second reaction of the COI-targeting snPCR from chicken skin were submitted under accession numbers KY025553 and KY025554, respectively.
